# Tracking *Alu *evolution in New World primates

**DOI:** 10.1186/1471-2148-5-51

**Published:** 2005-10-06

**Authors:** David A Ray, Mark A Batzer

**Affiliations:** 1 Department of Biological Sciences, Biological Computation and Visualization Center, Center for Bio-Modular Multiscale Systems, Louisiana State University, Baton Rouge, LA, 70803, USA; 2 Department of Biology, West Virginia University, Morgantown, WV, 26506, USA

## Abstract

**Background:**

*Alu *elements are Short INterspersed Elements (SINEs) in primate genomes that have proven useful as markers for studying genome evolution, population biology and phylogenetics. Most of these applications, however, have been limited to humans and their nearest relatives, chimpanzees. In an effort to expand our understanding of *Alu *sequence evolution and to increase the applicability of these markers to non-human primate biology, we have analyzed available *Alu *sequences for loci specific to platyrrhine (New World) primates.

**Results:**

Branching patterns along an *Alu *sequence phylogeny indicate three major classes of platyrrhine-specific *Alu *sequences. Sequence comparisons further reveal at least three New World monkey-specific subfamilies; *Alu*Ta7, *Alu*Ta10, and *Alu*Ta15. Two of these subfamilies appear to be derived from a gene conversion event that has produced a recently active fusion of *Alu*Sc- and *Alu*Sp-type elements. This is a novel mode of origin for new *Alu *subfamilies.

**Conclusion:**

The use of *Alu *elements as genetic markers in studies of genome evolution, phylogenetics, and population biology has been very productive when applied to humans. The characterization of these three new *Alu *subfamilies not only increases our understanding of *Alu *sequence evolution in primates, but also opens the door to the application of these genetic markers outside the hominid lineage.

## Background

SINEs (Short INterspersed Elements) are powerful tools for systematic and population biologists [1-8]. Examples of phylogenies elucidated using the SINE method include the use of SINEs to support the hypothesis that cetaceans (whales, dolphins and porpoises) form a clade within Artiodactyla [9], clarification of relationships between cichlid fishes [10-12] and the resolution of the human-chimpanzee-gorilla trichotomy [5]. Although applications of SINE elements to resolve population dynamics have been limited to humans [13-19] and, to a lesser extent, cichlid fishes [11,20,21], these studies have been very successful in revealing patterns of variation and there is every reason to believe that they can be as productively applied to other species.

One reason for the success of SINEs as phylogenetic and population genetic markers is that their mode of evolution is unidirectional [3,4,7,8,22]. This characteristic allows for a confident inference that the ancestral state is the absence of the SINE at each locus. Because there is no known mechanism for the specific removal of SINEs from any genome [4,23], individual SINEs are generally thought to be homoplasy-free characters [4,7,17,22-25].

*Alu *elements are primate-specific SINEs of ~300 bp. These elements have been extremely successful at propagating in primate genomes as evidenced by the fact that they make up ~10% of the human genome by mass [23,26]. Distinct subfamilies of *Alu *elements in the human genome have been described in detail [17,18,23,27-32]. Examination of these young subfamilies has provided us with clues as to the mobilization dynamics and evolution of *Alu *elements in the hominid lineage. Characterization of *Alu *mobilization in non-human primates has not been as complete. The ascertainment of lineage-specific subfamilies of *Alu *elements would increase our understanding of mobile element evolution in these organisms and allow for the development of SINE-based studies of population and evolutionary patterns.

We recently used *Alu *insertion loci to clarify various relationships among platyrrhine (New World monkeys, NWM) and cercopithecid (Old World monkeys) primates [33,34]. These projects produced examples of *Alu *insertions present in a wide variety of lineages along the primate tree. We have performed a phylogenetic analysis of the *Alu *sequences themselves (focusing on the platyrrhine-specific insertions) in order to characterize the evolutionary history of *Alu *lineages that have been or currently are retrotransposition competent in some non-human primates.

## Results and discussion

Platyrrhine-specific *Alu *sequences were obtained from the data sets used in Ray et al. [34] When available, the sequences from multiple taxa at a particular locus were aligned and a consensus sequence generated to create an approximation of the sequence of the original insertion. A total of 48 platyrrhine-specific insertions were collected. All selected sequences were examined for the presence of target site duplications (TSDs). The presence of these TSDs along with the absence of each marker in hominid and cercopithecid taxa (and from the genomes of other platyrrhine primates in many cases) serves to verify that the elements are the result of retrotransposition events and not segmental duplications. To trim potentially long branches and to verify the ability of the approach to recover previously established relationships among reference sequences, we added the consensus sequences for *Alu *elements specific to hominids (*Alu*Ya5, *Alu*Ya5a2, *Alu*Yb8, *Alu*Yb9, *Alu*Yc1, *Alu*Yc2, *Alu*Yd3, *Alu*Yd6, and *Alu*Ye5) [18,30-32,35-37]. We also included the canonical *Alu *consensus sequences for the Jb, Sc, Sg, Sp, Sq, Sx, and Y subfamilies [38-40] and rooted the tree on *Alu*Jb based on previously established relationships [40-42].

The methods used to identify informative loci among cercopithecid taxa primarily involved a linker-PCR strategy using two *Alu *selection primers [33]. Unfortunately, this introduced a sequence bias toward particular subfamilies of recently integrated or lineage specific *Alu *elements. The strategy used to identify informative platyrrhine loci, on the other hand, used a combined computational-experimental approach. Over half of the loci identified were derived from Bacterial Artificial Chromosome (BAC) sequences and thus no bias was introduced. In addition, a wide variety of primers was used in the linker-PCR approach; as a consequence, the bias was reduced for experimentally-derived loci. Because of the bias in the data derived from the cercopithecids, we have not included these *Alu *sequences in the analyses. For platyrrhine *Alu *lineages, however, more confident inferences can be made.

**Figure 1:**
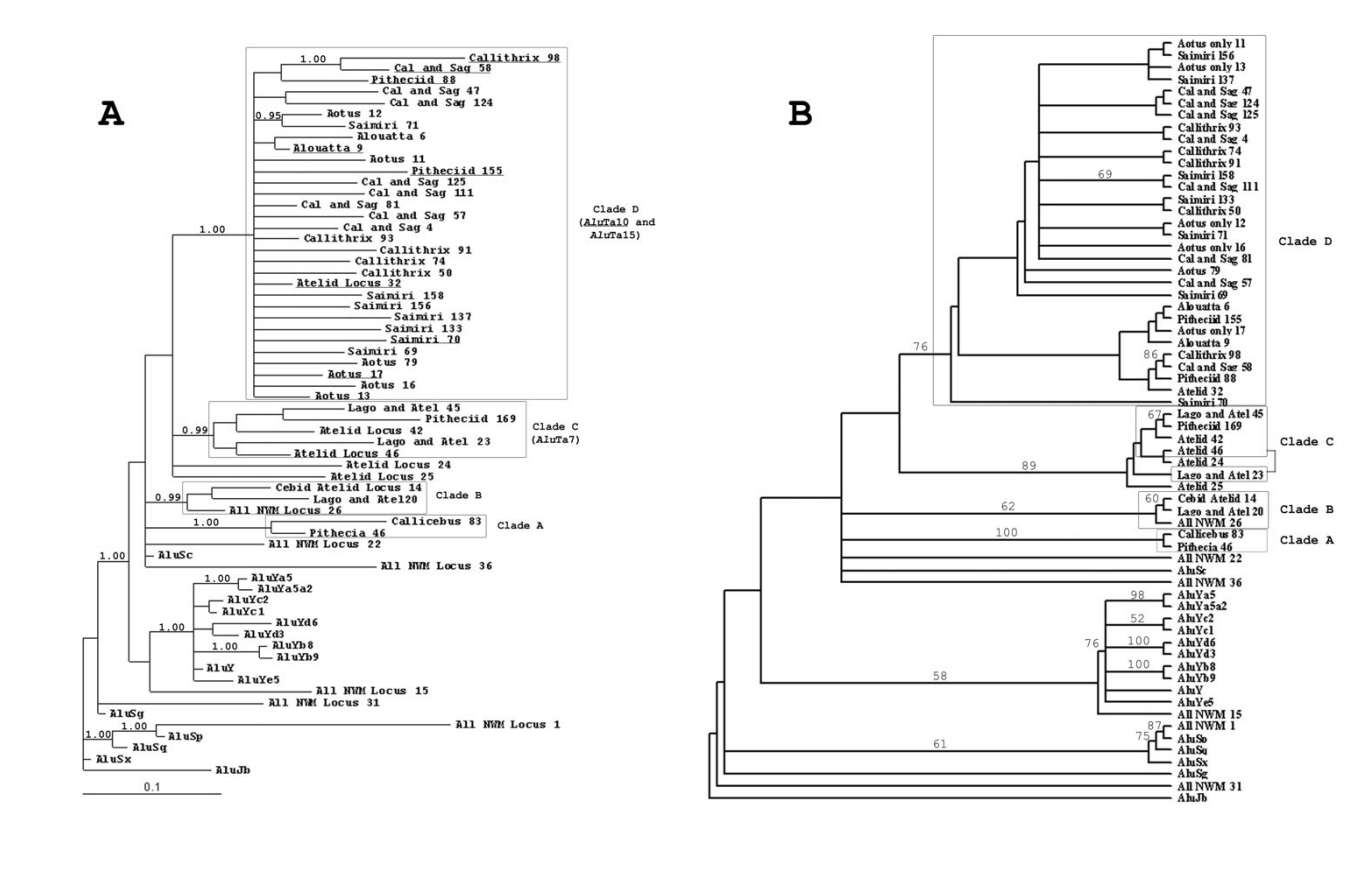
A) Majority-rules consensus of 10,000 trees generated using a Bayesian approach. Support values greater than 0.85 are indicated on relevant nodes. The major platyrrhine clades (A, B, C, and D) are indicated. Within clade D, members of subfamily *Alu*Ta10 are underlined. B) Majority-rules consensus tree of 107,470 equally parsimonious trees generated as described in the Methods section. Bootstrap values for nodes with greater than 50% support are indicated. Sequences representing well-supported clades from the Bayesian tree are also indicated.

Tree topologies recovered using Bayesian and parsimony criteria were generally congruent (Fig. 1). Minor differences in the placement of some sequences are observed but the well-supported clades recovered by the Bayesian analysis are often present in the parsimony consensus trees with reasonable support (>75%). However, bootstrap support on the parsimony-based cladogram was not as high for several of the major nodes found on the Bayesian tree. We suspect that this is due to the hybrid (partially gene converted) nature of 31 sequences that share diagnostic features of both *Alu*Sc-derived and *Alu*Sp derived elements (see below for a full discussion). Given the assumptions inherent in parsimony-based analyses (i.e. incremental sequence-based changes) hybrid elements that accumulated a whole suite of character states as a unit and that define other lineages in the data set would be expected to cause significant problems. Supplemental analyses with the hybrid elements removed confirmed this suspicion by raising support values at some nodes over 20 points (data not shown). The reduced tree-search method used is also thought to recover lower bootstrap support values than more traditional methods [43]. For these reasons, we have chosen to base our major conclusions on the topology and support values present on the Bayesian tree.

Within that tree, the established relationships between canonical *Alu *consensus sequences were recovered as expected. The *Alu*Jb subfamily is basal to the remaining *Alu *sequences and relationships between the various *Alu*S subfamilies are similar to the results of Kapitonov and Jurka [39]. Among the New World primate *Alu *sequences all but three platyrrhine-specific sequences fall within a well supported *Alu*Sc-*Alu*Y derived clade. This topology suggests that at there may have been three *Alu *lineages active at the time of the platyrrhine-catarrhine divergence around 35–40 million years ago [44]: an *Alu*Y progenitor; *Alu*Sp; and, *Alu*Sc. The three platyrrhine-specific *Alu *insertions that clustered outside the major platyrrhine *Alu*Sc/*Alu*Y-derived clade were 'All_NWM_Locus_1', 'All_NWM_Locus_15', and 'All_NWM_Locus_31'. Each of these insertions is present in all tested platyrrhine taxa, suggesting that they occurred before the radiation of the New World monkeys into three recognized families, (Cebidae, Atelidae and Pitheciidae). The *Alu *sequence at 'All_NWM_Locus_1' appears to be derived from an *Alu*Sp source gene. Direct observation of the *Alu *sequence confirms the presence of several *Alu*Sp diagnostic sites in this element (see supplemental alignments). Based on our analyses, the sequence for 'All_NWM_Locus_15' appears to be derived from an *Alu*Y progenitor. There is no significant support for the node, however, and it should be noted that this is merely a suggestion based on the topology of the tree. Thus, an *Alu*Y progenitor, *Alu*Sp, and *Alu*Sc were all active around the time of the split. The source of the sequence at 'All_NWM_Locus_31' is unclear given the differences in placement between the Bayesian and parsimony analyses. RepeatMasker [45] lists the element as belonging to the *Alu*Sg lineage. Thus, it may represent a fourth lineage that was active early in the evolution of New World monkeys.

A majority of the *Alu *sequences specific to various New World monkeys are most closely related to an *Alu*Sc and there are four well-supported clades within this group. Clade A is represented by two sequences that were found only in members of Pitheciidae. The insertions 'Callicebus_83' and 'Pithecia_46', were specific to their respective Pitheciid genera, and they share eight exclusive non-CpG mutations when compared to *Alu*Sc and other *Alu*Sc-like sequences (Bayesian support = 1.00). The close relationship between these sequences was also recovered in the parsimony analysis. While we will not assign them to a new subfamily based on only two sequences, we suggest that they are good candidates for a Pitheciid-specific lineage.

A second clade (B) within the putative *Alu*Sc-derived group was also highly supported (0.99) and was represented an insertion identified in all platyrrhine primates ('All_NWM_Locus_26'), as well as in two Atelid taxa ('Lago_and_Atel_20') and in all members of Cebidae and Atelidae ('Cebid_Atelid_Locus_14'). These sequences may represent an *Alu*Sc-derived subfamily. However, this cluster was based on only a few sequences and on shared mutations at CpG sites; thus, it should be interpreted cautiously. An alternative is that these and the other elements in this group represent true *Alu*Sc insertions that have continued to accumulate in platyrrhine genomes throughout their evolution. This is not unlikely given the recent observations of potentially polymorphic *Alu*Sc loci [46] and relatively recent *Alu*Sx insertions in humans [47]. The 'stealth' model of *Alu *evolution and dispersal reported by Han et al. [48] also predicts low levels of activity for older *Alu *subfamiles. *Alu*Sc may represent a hardy subfamily that has remained active at a low level for long periods of time in a variety of primate genomes.

Clade C (support = 0.99) comprises five sequences characterized by 11 shared mutations (including a 7-base duplication) that distinguish them from *Alu*Sc. Sequences in this clade are distributed among members of families Pitheciidae and Atelidae. One interpretation of this pattern is the emergence of the source gene prior to the expansion of a Pitheciid-Cebid clade but after the divergence of Atelid taxa. This hypothesis is unlikely, however, given the results of Ray et al. [34] in which it was made clear that family Pitheciidae was the first to diverge from the early platyrrhine groups. We suggest instead that the source gene emerged after the divergence of platyrrhine and catarrhine primates but before the platyrrhine radiation 17–20 mya [49,50], and that none of these elements was recovered for Cebid taxa due to sampling error. Additional work will be required to test this hypothesis.

Clade D is the largest of the clearly definable platyrrhine *Alu *clades, comprising 31 sequences from all three platyrrhine families. It is well-supported (1.00) and is distinguished by numerous shared mutations among its members. Of the new subfamilies described here, this lineage is particularly interesting because of its apparently unique origin. Close examination of the sequences reveals four shared *Alu*Sc diagnostic mutations at the 5' end of the elements; however, at the 3' end of the elements, there are five additional diagnostic sites characteristic of the *Alu*Sp subfamily. Examples of 'hybrid' elements have been described previously [17,25,29], but these represented individual instances involving the gene conversion of *Alu *elements already present in the genome. That does not appear to be the case here.

**Figure 2:**
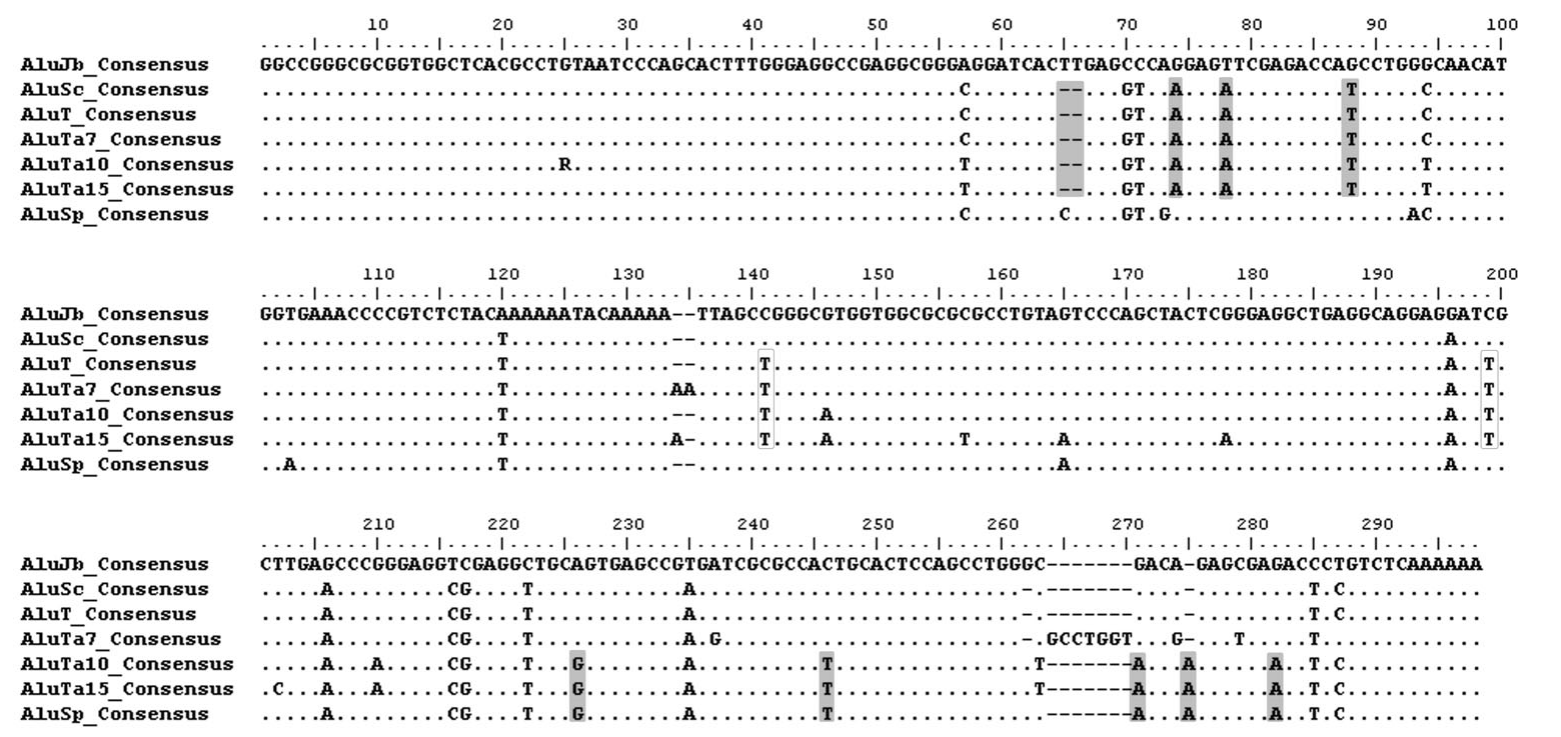
Multiple sequence alignment of three canonical reference sequences (*Alu*Jo, *Alu*Sc, and *Alu*Sp) with the new consensus sequences described in this work. Identical sequence residues are indicated by ".". Indels events are indicated by "-". Diagnostic mutations characteristic of *Alu*Sc and *Alu*Sp that are shared by the new consensus sequences are shaded. Substitutions distinguishing all *Alu*T subfamily members from *Alu*Sc are boxed.

The presence of 31 distinct elements harboring this combination of *Alu*Sc and *Alu*Sp diagnostic mutations (plus three additional shared mutations) suggests that there is a recently active source gene with these characteristics. We propose that a source gene (most likely derived from *Alu*Sc) existed early in platyrrhine primate evolution and that the 3' end of the element was subjected to a gene conversion event via any of the three potential models described by Kass et al. [51]. Starting somewhere between bases 199 and 226 and continuing to the end of the element, the conversion event resulted in the replacement of the sequence of the source gene with sequence from an *Alu*Sp-like element (Fig. 2). The result was a 'fusion' element that remained active and may still be active in the genomes of several platyrrhine primates.

This group of elements can be further subdivided into two subfamilies based on additional shared diagnostic mutations in what appears to be the more recently derived subfamily. In addition to the *Alu*Sp and *Alu*Sc derived sites and the three additional distinguishing sites, 21 elements share four unique mutations. Thus, clade D can be subdivided into two subfamilies consisting of 10 and 21 elements, respectively (see supplemental alignments).

These two subfamilies share two diagnostic positions with the previously mentioned clade C 5' to the appearance of the *Alu*Sp indicative sites. Thus, we believe that these three groups of sequences represent a new platyrrhine-specific subfamily we dubbed *Alu*T. We chose this designation based on the nomenclature proposed by Batzer et al. [38] in which younger subfamilies are assigned later letters of the alphabet. This is followed by a lowercase letter designating the order of publication, and a numerical designation indicating the number of diagnostic sites that differentiate it from the subfamily consensus. Because this group was similar to and apparently derived from *Alu*Sc, *Alu*T was most appropriate. It is distinguished from *Alu*Sc by the two aforementioned diagnostic mutations and can currently be divided into three subfamilies; *Alu*Ta7, *Alu*Ta10, and *Alu*Ta15 (Fig. 2). For reference, we have included a hypothetical *Alu*T consensus sequence based on the diagnostic sites shared by the Ta5, Ta10, and Ta15 consensi and the presumed ancestral sequence, *Alu*Sc, in figure 2.

Represented by 21 sequences, *Alu*Ta15 was only found in Cebid taxa (*Aotus*, *Callithrix*, and *Siamiri*). *Alu*Ta10 is represented by ten sequences and was recovered in members of all three platyrrhine families. The distribution of this subfamily of elements among platyrrine taxa and the pattern of shared diagnostic sites suggest that the *Alu*Ta10 family expanded earlier in platyrrhine evolution and may have given rise to the *Alu*Ta15 subfamily. A larger sample based solely on elements derived from unbiased methods will be required to test this hypothesis and is currently underway.

## Conclusion

The identification of three (potentially four) new subfamilies that are unique to platyrrhine primates represents a step forward in our understanding of the evolution of *Alu *elements in the genomes of non-hominid primates. Further, this is the first report of a unique mechanism of *Alu *subfamily generation. Until now, the evolution of *Alu *subfamilies could easily be described using the sequential accumulation of diagnostic mutations. For example, the hominid *Alu *subfamily *Alu*Yb currently consists of four variants, Yb7, Yb8, Yb9, and Yb11 [30,31,52]. Patterns of sequence variation clearly illustrate the hierarchical nature of sequence evolution in this family. Yb9 exhibits all of the diagnostic mutations defining *Alu*Yb7 and *Alu*Yb8 as well as its own signature mutation. *Alu*Yb11 follows suit by exhibiting all of the *Alu*Yb9 mutations plus two others. This pattern is confirmed using age estimates that suggest *Alu*Yb7 is the oldest and *Alu*Yb11 is the youngest. The *Alu*Ta10 and *Alu*Ta15 subfamilies represent the first documented cases of a recently active 'fusion' element in which the diagnostic mutations were not accumulated gradually over time; instead, they represent the sudden incorporation of several signature mutations by way of a gene conversion event. Thus, a new mechanism of *Alu *subfamily generation, though previously considered possible [29], has now been substantiated in the genome.

On a more practical level, a number of questions raised in other taxonomic analyses of New World monkeys can now be better addressed [1,34,53-60] given the data presented here. We can confidently assign subfamily status to certain individual *Alu *elements in platyrrhine genomes. Thus, we are able to target particular *Alu *subfamilies with known expansion timeframes to address branching patterns for particular primate lineages. This technique has previously proven valuable. For example, by combining a targeted analysis of the *Alu*Ye5 subfamily with sequence database searches for additional informative loci, we were able to confidently address the human-chimpanzee-gorilla trichotomy [5]. Application of similar techniques to other primates can easily be adapted by using the linker protocols described in Ray et al. [34], Xing et al. [33] and Roy et al. [61] and by computational analyses of existing sequence data.

At the population level, the amplification dynamics of *Alu *elements have been well characterized in humans and even in chimpanzees, but have not been investigated extensively in other primates. This is unfortunate given their utility in studies of genome evolution in humans and chimpanzees [62-64], population biology in humans [13,15,16,27,65-74], and phylogenetic analysis at all levels of the primate tree [2,5,6,33,34,41,75]. Knowledge of these subfamilies will aid in the development of markers useful for all of the above tasks. For example, given the endangered status of many New World taxa, the existence of easy-to-ascertain markers (via a single PCR) to identify species-specific *Alu *insertions in tissues of unknown origin will be a boon to conservation biologists and to population geneticists. Similar genetic systems have already proven useful in other taxa ranging from humans to waterfowl [76-78]. As one simple example, we now use many of the *Alu *loci used in this study to verify the identity of cell lines in our laboratory. Using a single PCR to amplify a taxon-specific Alu insertion is quick and efficient when compared to methods that involve morphological analysis (if possible on a tissue sample) or amplification and sequencing of DNA.

In this study we have identified diagnostic mutations for platyrrhine specific subfamilies. The identification of particular *Alu *lineages is the critical first step in identifying polymorphic elements in a primate taxon [17,18,31,61]. By identifying the subfamilies that are specific to particular taxa, researchers are now better able to use previously established techniques that take advantage of diagnostic mutations to identify useful markers at various taxonomic levels. The essentially homoplasy free nature of SINE markers makes them in some ways superior to other commonly used markers for population genetics [3,4,10,12,22,34]. Thus we see this as the beginning of a series of studies in which the SINE method of population genetic analysis will be expanded beyond our own species.

## Methods

Insertion/deletion (indels) events play a significant role in defining *Alu *subfamilies. For this reason, the phylogenetic method we used to reconstruct relationships was based primarily on the Bayesian method implemented by MrBayes, Ver. 3.1 [79,80]. We chose this method because of its robustness and its ability to take advantage of information present in the form of insertion/deletion events in the alignment. We partitioned the data into two sets, sequence data and gap data. For partition one, sequence parameters were estimated from the data The second partition was generated using indels that were present in two or more sequences. These were coded as present (sequence) or absent (gap). For the second data partition, we estimated rates of indel occurrence from the data and corrected for ascertainment bias by setting the coding option to 'variable' as per the MrBayes manual.

Two simultaneous Markov chain Monte Carlo analyses were performed using one cold and three heated chains (temperature set to default 0.2) for each analysis. We ran the analysis for 7.5 million generations, sampling the trees every 100 generations. At ~6.13. million generations, the standard deviation of split frequencies consistently reached a value of <0.01, indicating that both analyses had begun converging on similar trees. We discarded the first 6.5 million generations as burn-in and generated a majority-rules consensus tree. Nodes with probability values of 0.85 to 0.89 were considered to have low support, 0.90 to 0.94 to have moderate support and nodes greater than 0.95 to be highly supported [80].

As a comparison, we also performed a parsimony analysis of the data in PAUP* v4.0b10 [81]. Non-CpG dinucleotides were weighted at six times the value of CpG dinucleotides [82] and gaps were treated as a fifth character state. The size of the data set made a bootstrap analysis using a full heuristic search for each replicate impractical. For this reason, we employed a reduced tree-search bootstrapping method as described by DeBry and Olmstead [43] to ascertain support for nodes.

The sequences from each clearly defined clade (see Results and Discussion) were collected and examined for shared mutations that presumably represent diagnostic mutations or positions characteristic of mobile element subfamilies. Consensus sequences for each of these groups were constructed. For non-CpG sites, a simple majority-rules approach was taken to obtain the consensus for the site. *Alu *elements, however, are rich in CpG dinucleotides that are known to mutate at a 6-fold higher rate than non-CpG sites [82]. These sites tend to be highly variable and could represent a problem when determining the identity. We addressed this issue by examining types of variation at potential CpG sites and by referring to the presumed ancestral sequences. First, dinucleotide sites exhibiting high diversity that comprised primarily both CpA and TpG dinucleotides were assumed to be highly mutable CpG sites that decayed as the result of the spontaneous deamination of 5-methylcytosine. When it remained unclear whether or not the site should be considered a CpG dinucleotide, we referred to the *Alu*Sc or *Alu*Sp consensus sequences to determine the likely ancestral state for the site and made the appropriate assignment.

Sequence alignments used for phylogenetic analysis and for the generation of consensus sequences are available online as additional files.

## List of abbreviations

NWM – New world monkeys

Mya – million years ago

hLRT – hierarchical likelihood ratio test

AIC – Akaike Information Criterion

Indels – insertion/deletion events

## Authors' contributions

DAR initiated the study, collected and aligned all of the sequences used in the project, performed all analyses, interpreted the data, and prepared the manuscript. MAB provided input on the analysis and interpretation of the data and on all versions of the manuscript.

## Supplementary Material

Additional File 1 Multiple sequence alignment of all *Alu *sequences used in this study. Clades of sequences described in the text are delimited and the newly described consensus sequences are included. Click here for file
